# Safety of a killed oral cholera vaccine (Shanchol) in pregnant women in Malawi: an observational cohort study

**DOI:** 10.1016/S1473-3099(16)30523-0

**Published:** 2017-05

**Authors:** Mohammad Ali, Allyson Nelson, Francisco J Luquero, Andrew S Azman, Amanda K Debes, Maurice Mwesawina M'bang'ombe, Linly Seyama, Evans Kachale, Kingsley Zuze, Desire Malichi, Fatima Zulu, Kelias Phiri Msyamboza, Storn Kabuluzi, David A Sack

**Affiliations:** a Johns Hopkins Bloomberg School of Public Health, Baltimore, MD, USA; b Epidemiological Unit, Ministry of Health, Lilongwe, Malawi; c Johns Hopkins Project, Blantyre, Malawi; d World Health Organization, Malawi Country Office, Lilongwe, Malawi; e Preventive Health Services Department, Ministry of Health, Lilongwe, Malawi

## Abstract

**Background:**

Pregnancy increases the risk of harmful effects from cholera for both mothers and their fetuses. A killed oral cholera vaccine, Shanchol (Shantha Biotechnics, Hydrabad, India), can protect against the disease for up to 5 years. However, cholera vaccination campaigns have often excluded pregnant women because of insufficient safety data for use during pregnancy. We did an observational cohort study to assess the safety of Shanchol during pregnancy.

**Methods:**

This observational cohort study was done in two adjacent districts (Nsanje and Chikwawa) in Malawi. Individuals older than 1 year in Nsanje were offered oral cholera vaccine during a mass vaccination campaign between March 30 and April 30, 2015, but no vaccines were administered in Chikwawa. We enrolled women who were exposed to oral cholera vaccine during pregnancy in Nsanje district, and women who were pregnant in Chikwawa district (and thus not exposed to oral cholera vaccine) during the same period. The primary endpoint of our analysis was pregnancy loss (spontaneous miscarriage or stillbirth), and the secondary endpoints were neonatal deaths and malformations. We evaluated these endpoints using log-binomial regression, adjusting for the imbalanced baseline characteristics between the groups. This study is registered with ClinicalTrials.gov, number NCT02499172.

**Findings:**

We recruited 900 women exposed to oral cholera vaccine and 899 women not exposed to the vaccine between June 16 and Oct 10, 2015, and analysed 835 in each group. 361 women exposed to the vaccine and 327 not exposed to the vaccine were recruited after their pregnancies had ended. The incidence of pregnancy loss was 27·54 (95% CI 18·41–41·23) per 1000 pregnancies among those exposed to the vaccine and 21·56 (13·65–34·04) per 1000 among those not exposed. The adjusted relative risk for pregnancy loss among those exposed to oral cholera vaccine was 1·24 (95% CI 0·64–2·43; p=0·52) compared with those not exposed to the vaccine. The neonatal mortality rate was 11·78 (95% CI 5·92–23·46) per 1000 livebirths for infants whose mothers were exposed to oral cholera vaccine versus 8·91 (4·02–19·77) per 1000 livebirths for infants whose mothers were not exposed to the vaccine (crude relative risk 1·32, 95% CI 0·46–3·84; p=0·60). Only three newborn babies had malformations, two in the vaccine exposure group and one in the no-exposure group, yielding a relative risk of 2·00 (95% CI 0·18–22·04; p=0·57), although this estimate is unreliable because of the small number of outcomes.

**Interpretation:**

Our study provides evidence that fetal exposure to oral cholera vaccine confers no significantly increased risk of pregnancy loss, neonatal mortality, or malformation. These data, along with findings from two retrospective studies, support use of oral cholera vaccine in pregnant women in cholera-affected regions.

**Funding:**

Bill & Melinda Gates Foundation.

## Introduction

Pregnant women with cholera are at risk of complications, leading to fetal losses in 2–36% of cases if not treated promptly.^1^, ^2^ Killed, whole-cell, oral cholera vaccines are recommended by WHO to reduce the risk of cholera. Licensed oral cholera vaccines include Dukoral (Valneva, Lyon, France), Shanchol (Shantha Biotechnics, Hydrabad, India), mORCVAX (Vabiotech, Hanoi, Vietnam), and Euvichol (EuBiologic Co, Ltd, Chuncheon, South Korea). Shanchol is used most commonly in outbreak response in low-income countries, and has a cumulative efficacy of 65% over 5 years.^3^ Shanchol and Euvichol are available through the global stockpile of oral cholera vaccine.^4^ Findings from clinical trials with non-pregnant participants have shown that oral cholera vaccine is safe.^5^ However, cholera vaccination campaigns often exclude pregnant women because of insufficient data about the safety of the vaccine during pregnancy. WHO recommends vaccination of pregnant women in cholera-endemic settings, for whom the risk of cholera infection can be high.^6^ The package inserts for Dukoral and Shanchol are cautious about the use of these vaccines during pregnancy, because definitive evidence of safety during pregnancy is not available. In Tanzania, the risk of pregnancy loss was not significantly higher among pregnant women who were inadvertently vaccinated with Dukoral during the mass vaccination campaign in 2009.^7^ Findings from a retrospective cohort study in Guinea showed no evidence of increased risk of pregnancy loss after receiving Shanchol.^8^ However, such retrospective studies are subject to biases and represent low-quality evidence.

**Research in context**

**Evidence before this study**

Many cholera vaccination campaigns have excluded pregnant women based on package inserts stressing the lack of information about safety in pregnant women. We searched PubMed on July 24, 2016, with no date or language restrictions, for reports about cholera vaccine in pregnant women with the terms “cholera vaccine” AND “pregnant women” and without filtering any other information, which resulted in only two relevant publications. In the first study, from Zanzibar, oral cholera vaccination with Dukoral during a mass vaccination campaign in 2009 did not cause any harmful effects on pregnancies. The surveillance for detecting adverse pregnancy outcomes in the study was done 9 months after vaccination. Findings from a second study done in Guinea also showed no evidence of increased risk of pregnancy loss after receiving Shanchol. This study was a retrospective cohort study assessing pregnancy outcomes among vaccinated and unvaccinated women who were pregnant at the time of vaccination and among those who became pregnant after vaccination; hence, their fetuses were not exposed to oral cholera vaccine. Both of these studies were retrospectively documented; no prospective studies with systematic follow-up have been done to assess the safety of oral cholera vaccine when given to pregnant women, and therefore safety concerns about oral cholera vaccination in pregnant women have persisted. This existing body of data suggested that more evidence is needed to inform decisions about inclusion of pregnant women in cholera vaccination programmes.

**Added value of this study**

In our study we noted no evidence of increased pregnancy loss after oral cholera vaccination during pregnancy. Our findings are consistent with those from the two earlier retrospective studies, and provide evidence that exposure to oral cholera vaccine is not associated with increased risk for neonatal death or newborn malformation.

**Implications of all the available evidence**

Women in endemic settings are at high risk of diarrhoeal diseases, and contraction of severe diarrhoeal disease such as cholera during pregnancy can result in pregnancy loss, premature childbirth, or maternal death if the patient is not treated properly. According to WHO, individuals who are at risk of severe disease and for whom vaccines are not contraindicated should also be targeted by killed oral cholera vaccine. Findings from our analysis, along with results from previous studies, strongly support inclusion of pregnant women during cholera vaccine campaigns because there is no evidence of a harmful effect on the fetus and these women will benefit from the reduced risk of the disease; their exclusion from vaccination campaigns puts them at risk. This recommendation is consistent with recommendations for other non-live vaccines during pregnancy (eg, tetanus toxoid and injectable influenza).

On Jan 13, 2015, Malawi declared a state of disaster after widespread floods. Between Feb 11 and June 21, 2015, 693 cases of cholera occurred, with 11 deaths in eight districts representing a case-fatality rate of 1·6%.^9^ 599 (86%) of 693 cases and seven (64%) of 11 deaths were from the two districts, Nsanje and Chikwawa.

A mass cholera vaccination campaign was initiated on March 30, 2015, by the Ministry of Health, providing two doses of Shanchol to individuals aged 1 year or older, irrespective of their pregnancy status. The decision of the Malawi Ministry of Health to include pregnant women was made in consultation with WHO and other implementing partners, based on WHO recommendations.^6^ Oral cholera vaccine was provided to the residents of Nsanje but not the adjacent district Chikwawa because of insufficient supply of the vaccine.^10^ 156 592 people received the first dose of oral cholera vaccine and 108 237 received two doses of the vaccine.^10^ This study evaluates the pregnancy outcomes of women in Nsanje who received the vaccine while pregnant compared with those who were pregnant in Chikwawa at the start of the vaccination campaign and did not receive the vaccine.

## Methods

### Study design and participants

In this observational cohort study, we recruited women who received at least one dose of the oral cholera vaccine and who were pregnant at the time of vaccination (ie, whose fetuses were exposed to oral cholera vaccine), and women who did not receive oral cholera vaccine until their time of delivery and who were pregnant on March 30, 2015.

**Figure 1:**
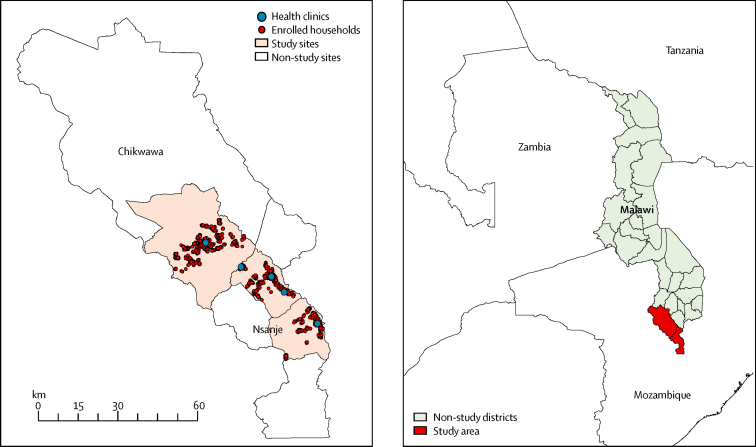
Spatial distributions of the enrolled women in the study area Digital maps were obtained from DIVA-GIS.

The study was done in two neighbouring districts in the southern region of Malawi, Nsanje and Chikwawa (figure 1). Malawi has one of the highest maternal mortality ratios in the world (634 per 100 000 livebirths).^11^ Miscarriage is estimated to occur in about 15% of all recognised pregnancies.^12^ The stillbirth rate was estimated to be 24 per 1000 births in 2009,^13^ and the neonatal mortality rate was estimated to be 27 per 1000 livebirths from 2011 to 2016.^14^

We recruited pregnant women from Nsanje who had their last menstrual period at least 3 weeks before their first dose of oral cholera vaccine and who received at least one dose, and women from Chikwawa who had their last menstrual period at least 3 weeks before March 30, 2015 (the start of the vaccination campaign), but who did not receive any doses of oral cholera vaccine on March 30, 2015.

Trained study staff obtained written informed consent or assent from all study participants in the local language. For women who were not literate, the study staff read the entire consent form and participants were permitted to use their fingerprint in place of their signature; an impartial witness verified the consent. Unmarried women younger than 18 years were included after receipt of consent from their parent or legal guardian. Ethical clearance and oversight was provided by the Johns Hopkins Bloomberg School of Public Health institutional review board and the National Health Sciences Research Committee of Malawi.

### Procedures

Before enrolment, community meetings were organised where the objectives of the study were explained to community leaders and local health and administration officials. Recruitment visits were made between June 16 and Oct 10, 2015. Initially, female village health volunteers (approximately one per village) enumerated the households in their villages and identified the women who were pregnant at any point between March 30 and April 30, 2015. Using this list, 16 trained interviewers who had completed a 4-day training programme (including a primer on human research) visited the households of women with identified pregnancies. Household visits continued until the desired sample size was reached.

After enrolment, the study staff collected information about sociodemographic characteristics, current pregnancy, obstetric history, cholera vaccination status, and the global positioning satellite location of the household. Vaccination status was verified by vaccination card or entry in the vaccination register. For women who had not delivered by the time of enrolment, pregnancy status was determined by either documentation of gestational age and due date in their National Health Passport and visible signs of pregnancy, or a pregnancy test. Pregnancy tests were done at the household and the test result was individually communicated to the enrolled women. For women whose pregnancies ended between March 30, 2015, and the date of enrolment, we collected additional information including date of delivery, type of outcome, and the risk factors related to their pregnancy.

The village health volunteers conducted monthly home visits to determine pregnancy status. After delivery, study staff visited the woman to collect information about delivery outcome and the health of the newborn baby for livebirths. All newborn babies presenting with health problems, per mothers' reports, were referred to the designated health facility where a qualified medical professional did a medical examination to detect and manage any malformations or other health issues. Clinicians at these facilities caring for these babies completed a standard questionnaire.

### Outcomes

The primary endpoint was pregnancy loss (spontaneous miscarriage or stillbirth) among women exposed to oral cholera vaccine while they were pregnant compared with women not exposed but who were pregnant during the same period. We defined spontaneous miscarriage as the expulsion of an embryo or fetus from its mother at 20 weeks' gestation or earlier.^7^ We defined stillbirth as a pregnancy loss which occurred on or after 21 weeks of gestation.^15^

The secondary endpoints were neonatal deaths and malformations. Neonatal deaths were defined as deaths occurring within 28 days of delivery. A malformation was defined as a physical defect in a live infant that was identified by a clinician at designated health facilities. Because this study was observational, we developed a clinical examination form that included a screening process to detect abnormalities, and then referred the children with abnormalities to the routine government health-care system to establish a diagnosis and manage the health issue.

### Statistical analyses

We estimated that 800 women exposed to oral cholera vaccine and 800 women not exposed to oral cholera vaccine would be necessary to have 80% power to detect a 1·5-times increase in the risk of pregnancy loss among vaccinated women with α of 0·05. We assumed that 7% of pregnancies would result in pregnancy loss in unvaccinated women. Assuming 10% loss to follow-up, our target sample size was 900 in each group.

We compared the occurrence of pregnancy loss, neonatal death, and newborn malformation between the two groups. We compared individual-level baseline variables judged to be potentially related to the risk of pregnancy loss between the two groups using χ^2^ or Fisher's exact tests when expected values in any of the cells of a contingency table are below 5, for categorical variables and Student's *t* or Mann-Whitney *U* tests for continuous variables. In a crude analysis, we estimated the rate ratio of outcomes in women exposed versus those not exposed to oral cholera vaccine using a generalised linear model, assuming a binomial family and a link log (log-binomial regression). In a multivariable model, we adjusted the rate ratio for significantly imbalanced baseline characteristics between the two groups. We followed the rule of ten events per covariate to maximise the coverage of the confidence interval of the estimate from a regression model.^16^ We classified associations as statistically significant if p was less than 0·05. Data were analysed with SAS version 9.3.

This study is registered at ClinicalTrials.gov, number NCT02499172.

### Role of the funding source

The sponsor of the study had no role in study design, data collection, analysis, or interpretation, or writing of the report. The corresponding author had full access to all the data in the study and had final responsibility for the decision to submit for publication.

## Results

**Figure 2:**
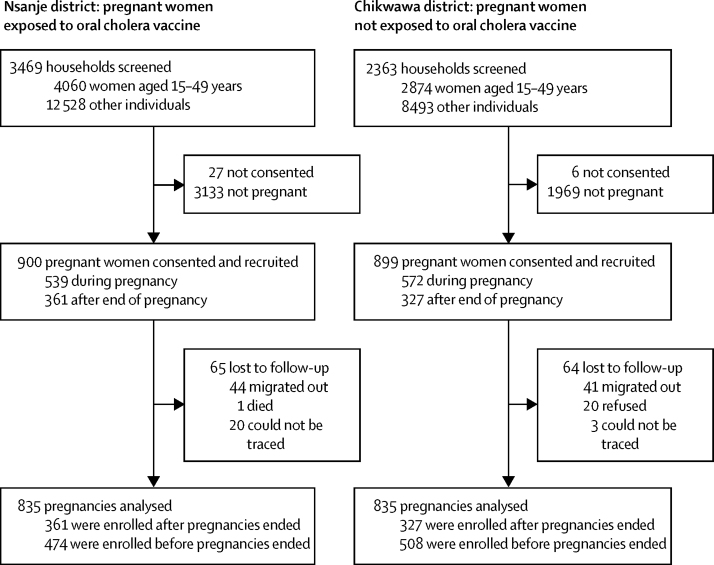
Study design

We recruited 900 women who were exposed to oral cholera vaccine in Nsanje district and 899 women who were not exposed to oral cholera vaccine in Chikwawa district; 65 women in the exposure group and 64 in the no-exposure group were lost to follow-up (figure 2). 361 women exposed to the vaccine and 327 not exposed to the vaccine were recruited after their pregnancies had ended.

The baseline characteristics of enrolled women between the groups were very similar, with the exception of availability of electricity in the house, consumption of tea or coffee during pregnancy, consumption of illegal drugs during pregnancy, and distance to the nearest heath facility (table 1). By contrast, among those who were lost to follow-up, age and distance to the nearest heath facility significantly differed between the two groups (appendix), with women in Nsanje district who were exposed to the vaccine being older and closer to the nearest health facilities than their counterparts in Chikwawa district who were not exposed to the vaccine. 1631 (98%) of 1670 women included in the analysis made at least one antenatal care visit at a health facility.

401 (48%) of 835 women who received the oral cholera vaccine received two doses; the rest received only one dose. Among women exposed to oral cholera vaccine, 228 (27%) were exposed to the first dose during their first trimester (0–13 weeks of gestational age), 334 (40%) during their second trimester (14–26 weeks of gestational age), and 273 (33%) during their third trimester (27–40 weeks of gestational age). Mean gestational age at the start of the vaccination campaign was 19·75 weeks (SD 9·81) among women exposed to the vaccine and 19·39 (SD 9·46) weeks among women not exposed to the vaccine (p=0·45; table 1).

One maternal death occurred (in the vaccine exposure group), resulting in an estimated maternal mortality ratio of 61 per 100 000 livebirths (95% CI 8–436) across the entire study population. There were 812 livebirths, six miscarriages, and 17 stillbirths among women who were exposed to the oral cholera vaccine compared with 817 livebirths, two miscarriages, and 16 stillbirths among women who did not receive the oral cholera vaccine. Less than 1% of pregnancies resulted in miscarriage and the stillbirth rate was 19·76 per 1000 births (95% CI 14·09–27·70) in the total study sample. The overall frequency of pregnancy loss was 27·54 (95% CI 18·41–41·23) per 1000 pregnancies in women exposed to cholera vaccine and 21·56 (13·65–34·04) per 1000 pregnancies in women not exposed to the vaccine. The relative risk of pregnancy loss after exposure to oral cholera vaccine was 1·24 (95% CI 0·64–2·43; p=0·52) after controlling for the factors found to be imbalanced between the two arms of the study.

We evaluated neonatal mortality among the 679 births in the vaccine exposure group and 673 births in the no-exposure group that were visited at least 29 days after delivery, plus deaths that were recorded during visits before 29 days. We noted eight deaths in the vaccine exposure group and six deaths in the no-exposure group. We estimated a neonatal mortality rate among infants whose mothers were exposed to oral cholera vaccine of 11·78 (95% CI 5·92–23·46) per 1000 livebirths, compared with 8·91 (4·02–19·77) per 1000 livebirths for infants whose mothers were not exposed to the vaccine. In the crude analysis, the risk for neonatal death was 1·32 (95% CI 0·46–3·84; p=0·60) among women exposed to oral cholera vaccine compared with that among women not exposed (table 2).

23 children from the vaccine exposure group and 12 children from the no-exposure group were referred to a clinician for a health assessment. After clinical examination, two newborn babies in the vaccine exposure group and one baby in the no-exposure group were considered to have a malformation (relative risk 2·00 [95% CI 0·18–22·04; p=0·57] for vaccine exposure compared with no exposure). These malformations included one infant with congenital limb defect, one with Down's syndrome, and one not growing normally.

In post-hoc analyses, we disaggregated pregnancy loss into stillbirth and spontaneous miscarriage. The adjusted relative risk of stillbirth among the exposure group was 1·17 (95% CI 0·56–2·44; p=0·68), and the crude estimate of risk for spontaneous miscarriage was 3·01 (95% CI 0·61–14·98; p=0·18).

## Discussion

In this observational cohort study, we noted no evidence that oral cholera vaccine during pregnancy significantly increased pregnancy loss, consistent with findings from earlier retrospective studies.^7^, ^8^ Our results also extend the observation that exposure to oral cholera vaccine while pregnant is not associated with an increased risk for neonatal death or newborn malformation. However, the number of malformations is too small to derive firm conclusions.

Cholera causes miscarriages and stillbirths in pregnant women.^17^ In a study done in the 1960s in Bangladesh,^18^ half of pregnant patients with cholera lost their fetuses in the third trimester of pregnancy. Fetal losses among women with cholera in their second or third trimester of pregnancy have varied in later studies, from 6% in two different studies in Peru in 1991,^19^, ^20^ to 12% in Senegal in 2006^2^, to 8% in a more recent study in Haiti.^1^ In our study, we noted 23 pregnancy losses among women exposed to oral cholera vaccine and 18 pregnancy losses among women not exposed to the vaccine. Although not statistically significant, we noted a slightly higher risk of pregnancy loss among women exposed to the vaccine. However, the potential additional risk of pregnancy loss associated with vaccine was very small (<1%). This hypothetical risk needs to be considered in the context of substantial risk of disease for the mother during an outbreak and a high risk of pregnancy loss (6–12%) if cholera does occur.^1^, ^2^, ^19^, ^20^

In our study, women who received oral cholera vaccine during their pregnancy were similar to those who did not receive the vaccine in terms of baseline sociodemographic characteristics and pregnancy history, with the exception of a few baseline characteristics (appendix). The significantly increased distance from household to the nearest heath facility among women in Chikwawa district (who were not exposed to oral cholera vaccine) compared with women in Nsanje district (who were exposed to the vaccine) suggests that distance to the nearest health facility did not affect risk of pregnancy loss in those communities.

We noted a lower maternal mortality rate in our study than the national estimate. This discrepancy might have resulted from the monthly follow-up visits that our participants received, and encouraging women to seek early antenatal care and to deliver in a health facility. Roughly 15% of pregnancies are estimated to result in miscarriage in Malawi; but we observed only eight miscarriages (incidence <1%). The unusually low number is probably due to our enrolment procedure, because we enrolled women at various gestational ages and therefore some miscarriages in the first trimester were not captured. Additionally, because there is no common system for the classification of miscarriage, we set 20 weeks as the standard instead of 24 weeks^21^ to allow comparison of our results with those from a previous study about the safety of oral cholera vaccine in pregnant women.^7^ If early stillbirths (21–28 weeks) were included in the miscarriage definition, per WHO guidance, our data show that approximately 1% of pregnancies ended in miscarriage.

The main strength in our study is the prospective design with large samples. Past studies that investigated safety of oral cholera vaccine in pregnant women were fully retrospective in nature,^7^, ^8^ and inherently incorporated telescoping bias, which is not the case for our study. Another strength is that the exposed and unexposed groups were very similar; we recruited both groups of women at the same time and could follow them up systematically until conclusion of their pregnancies, and thus the data from our study are free from seasonal bias between the two groups of women. Finally, we recruited women who were documented to have received vaccine on the basis of their vaccination cards or whose names could be verified in the vaccination register, and thus there was little risk of misclassification of vaccination status.

The main limitation in our study is the relative paucity of data about possible miscarriage during the first trimester; we were therefore unable to fully characterise the risk of pregnancy loss in the first trimester after oral cholera vaccination. The lower-than-expected number of miscarriages reduced the proportion of pregnancies that ended in fetal loss in our study. However, because the distribution of gestational ages at enrolment did not significantly differ between the two groups, our risk estimate for pregnancy loss due to exposure to oral cholera vaccine is not affected by this decreased proportion of pregnancy loss.

The second limitation of our study is that about 40% of enrolled women were recruited after their deliveries, and therefore information about their pregnancy outcomes was retrospectively collected. The mean time between delivery and data collection for these participants was 6 weeks. A third limitation was the inability to assess the health status of all newborn babies immediately after delivery. Therefore, the self-reported health assessment of the newborn babies could be subject to bias of delayed assessment. However, this limitation did not affect our estimation of frequency of our primary outcome.

Finally, this study was not an individually randomised, placebo-controlled clinical trial, which is generally considered to be the optimal study design. Rather, it compared pregnancy outcomes in adjacent districts in groups exposed or not exposed to oral cholera vaccine. Such a placebo-controlled clinical trial was not possible either logistically or ethically. Because oral cholera vaccination is now recommended by WHO, we did not think it ethical to randomly assign placebo to pregnant women during a cholera outbreak, because it would have deprived them of the benefit of an efficacious vaccination during a time when they were at high risk of cholera. Logistically, determination of the pregnancy status of women coming for vaccination would not have been possible during the campaign, especially in those who might have been in their first trimester and possibly not aware of their pregnancy.

A large body of evidence supports the safety of immunisation in pregnancy with non-live vaccines such as oral cholera vaccine.^22^, ^23^ No vaccine is licensed for use specifically during pregnancy, and to our knowledge no efficacy or immunogenicity studies in pregnant women have been done. In addition to the clinical information from our study and previous studies of oral cholera vaccine during pregnancy,^7^, ^8^ there are other reasons to consider the vaccine safe. First, the bacteria in the vaccines are killed and cannot replicate; thus, they are not able to cause illness themselves. Second, the vaccine antigens act locally on the gastrointestinal mucosa; thus, these antigens are unlikely to cause any systemic toxicity.^24^ Also, several other killed vaccines (eg, tetanus toxoid and injectable influenza) are provided routinely to pregnant women, even though their safety was not established during prelicensure testing.^22^, ^23^ Thus, on the basis of current understanding of the vaccine and evidence from our clinical studies,^7^, ^8^ pregnant women should not be excluded from oral cholera vaccination during vaccine campaigns. They would be at high risk if they develop cholera and this risk can be lowered with oral cholera vaccine without clinically significant risk to the woman or her fetus.

For **DIVA-GIS** see http://diva-gis.org

## Figures and Tables

**Table 1:** Baseline characteristics

	**Women exposed to oral cholera vaccine (n=835)**	**Women not exposed to oral cholera vaccine (n=835)**	p value*
At least primary schooling	295 (35%)	279 (33%)	0·40
Electricity in the house	60 (7%)	36 (4%)	0·0116
Owns house	745 (89%)	756 (91%)	0·37
Drank coffee or tea during pregnancy	396 (47%)	610 (73%)	<0·0001
Drank alcohol during pregnancy	9 (1%)	7 (1%)	0·61
Took illegal drugs during pregnancy	20 (2%)	6 (1%)	0·0057
Smoked cigarettes during pregnancy	4 (<1%)	3 (<1%)	0·70
Had history of pregnancy loss	139 (17%)	123 (15%)	0·28
Enrolled after delivery	361 (43%)	327 (39%)	0·09
Age (years)	25·93 (6·51)	25·75 (6·81)	0·58
Linear distance from household to the nearest health-care facility (km)	3·60 (3·57)	5·62 (3·38)	<0·0001
Gestational age at enrolment (weeks)	34·72 (10·16)	33·94 (9·69)	0·11
Gestational age at the start of vaccination (March 30, 2015) (weeks)	19·75 (9·81)	19·39 (9·46)	0·45

Data are n (%) or mean (SD).

* p values derived from χ^2^ test for categorical variables and *t* test for continuous variables.

**Table 2:** Risk of adverse outcomes in women exposed to oral cholera vaccine in simple and multivariable models

	**Women exposed to oral cholera vaccine**			**Women not exposed to oral cholera vaccine**			**Crude estimate**		**Adjusted estimate**	
	Total samples	Number of cases	Incidence per 1000 (95% CI)	Total samples	Number of cases	Incidence per 1000 (95% CI)	Relative risk (95% CI)	p value	Relative risk (95% CI)	p value
Pregnancy loss	835*	23	27·54 (18·41–41·23)	835*	18	21·56 (13·65–34·04)	1·28 (0·69–2·40)	0·43	1·24 (0·64–2·43)†	0·52
Stillbirth	835	17	20·36 (12·72–32·59)	835	16	19·16 (11·79–31·13)	1·06 (0·53–2·12)	0·86	1·17 (0·56–2·44)‡	0·68
Spontaneous miscarriage§	835	6	7·18 (3·24–15·95)	835	2	2·39 (0·60–9·56)	3·01 (0·61–14·98)	0·18	..	..
Neonatal death§	679¶	8	11·78 (5·92–23·46)	673¶	6	8·91 (4·02–19·77)	1·32 (0·46–3·84)	0·60	..	..
Malformation§	822‖	2	2·43 (0·61–9·70)	823‖	1	1·22 (0·17–8·62)	2·00 (0·18–22·04)	0·57	..	..

* Number of pregnancies.

† Adjusted for illegal drug use, consumption of tea or coffee, and distance from household to the nearest treatment centre (three most significantly different characteristics).

‡ Adjusted for consumption of tea or coffee and distance from household to the nearest treatment centre.

§ Adjusted estimate not calculated because there were too few events for the adjusted model.

¶ Excluded alive children visited before 28 days after delivery.

‖ Number of livebirths.
