# Supplementary material for: Safety of a killed oral cholera vaccine (Shanchol) in pregnant women in Malawi: an observational cohort study
Source: Lancet Infect Dis. 2017 May;17(5):538–44. doi: 10.1016/S1473-3099(16)30523-0 (PMC5406486; doi:10.1016/S1473-3099(16)30523-0)
Supplement: Supplementary appendix [file mmc1.pdf]

THE LANCET
Infectious Diseases

Supplementary appendix

This appendix formed part of the original submission and has been peer reviewed. We post it as supplied by the authors.

Supplement to: Ali M, Nelson A, Luquero FJ, et al. Safety of a killed oral cholera vaccine (Shanchol) in pregnant women in Malawi: an observational cohort study. *Lancet Infect Dis* 2017; published online Feb 1. http://dx.doi.org/10.1016/S1473-3099(16)30523-0.

Table S1. Baseline characteristics between women exposed and not exposed to OCV and who were loss to follow-up

Baseline characteristics	Exposed to OCV (n=65)	Not exposed to OCV (n=64)	P-value*
At least primary schooling	22 (33.85)	22 (34.38)	0.95
Electricity in the house	3 (4.62)	4 (6.25)	0.68
Owns house	55 (84.62)	52 (81.25)	0.61
Drank coffee/tea during pregnancy	0 (0.00)	0 (0.00)	-
Drank alcohol during pregnancy	0 (0.00)	0 (0.00)	-
Took illegal drugs during pregnancy	0 (0.00)	0 (0.00)	-
Smoked cigarettes during pregnancy	0 (0.00)	0 (0.00)	-
Had past history of pregnancy loss	15 (23.08)	9 (14.06)	0.19
Average age of the women	27.15 (7.26)	24.56 (6.69)	0.04
Average distance (linear) from household to the nearest health care facility (km)	3.94 (3.65)	5.28 (2.89)	0.02
Average gestational age at enrollment (weeks)	27.64 (7.46)	26.84 (6.15)	0.51
Average gestational age at the start of vaccination (March 30, 2015) (weeks)	12.81 (7.48)	12.38 (5.99)	0.72

* The p-values were derived from chi-square test for the categorical variables and from t-test for the dimensional variables

Table S2. Risk factors for pregnancy loss in a multivariable model

Variables	Relative risk	95% CI	P-value
Exposed to OCV	1.245	0.638-2.429	0.5205
Drank coffee/tea during pregnancy	0.652	0.340-1.247	0.1957
Took illegal drugs during pregnancy	1.740	0.227-13.347	0.5940
Distance from household to the nearest health care facility	1.051	0.972-1.136	0.2131
